# Morphological Changes in Endothelial Cell Organelles in a No-Touch Saphenous Vein Graft

**DOI:** 10.21470/1678-9741-2022-0111

**Published:** 2022

**Authors:** Akira Sugaya, Nobuhiko Ohno, Takashi Yashiro, Koji Kawahito

**Affiliations:** 1 Department of Cardiovascular Surgery, School of Medicine, Jichi Medical University, Shimotsuke, Tochigi, Japan; 2 Department of Anatomy, Division of Histology and Cell Biology, School of Medicine, Jichi Medical University, Shimotsuke, Tochigi, Japan; 3 Division of Ultrastructural Research, National Institute for Physiological Sciences, Myodaiji-cho, Okazaki, Aichi, Japan; 4 Nasu School of Nursing, Nasushiobara, Tochigi, Japan; 5 Faculty of Medicine, Universitas Muhammadiyah Prof. Dr. Hamka, Parung Serab, Kec. Ciledug, Kota Tangerang, Indonesia

**Keywords:** Coronary Artery Bypass, Saphenous Vein, No-Touch Saphenous Vein Graft, Transmission Electron Microscopy, Organelle.

## Abstract

**Introduction:**

Improved long-term patency of the no-touch (NT) saphenous vein graft has been reported to result from the preservation of a healthy vascular microstructure, especially endothelial cells. However, the precise morphology of endothelial cells and their organelles in NT saphenous vein graft has not been fully investigated. In this study, we assessed the ultrastructure of preserved endothelial cells in saphenous vein graft using transmission electron microscopy.

**Methods:**

Intact control (IC) vein, NT saphenous vein graft, and conventional (CT) saphenous vein graft were harvested from a patient. After observation by light microscopy, the nuclei and mitochondria in the preserved endothelial cells were compared among IC, NT, and CT using transmission electron microscopy, and the endothelial organelles were assessed quantitatively.

**Results:**

Light microscopy showed that the preservation of endothelial cells was comparable in IC, NT, and CT. Subsequent transmission electron microscopy observation showed that the nuclei in preserved endothelial cells appeared more swollen in CT than that in NT. Quantitative analysis revealed that nuclear size and circularity of preserved endothelial cells in NT and IC were similar, but those in CT were larger and higher, respectively, than those in IC and NT. In addition, the mitochondrial size in preserved endothelial cells in CT was larger than that in IC and NT.

**Conclusion:**

Necrotic changes in endothelial organelles characterized by swelling of nuclei and mitochondria were prominent in CT saphenous vein graft. The normally maintained ultrastructure of preserved endothelial cells in NT saphenous vein graft could contribute to long-term patency.

**Table:** 

Abbreviations, Acronyms & Symbols	
CABG	= Coronary artery bypass grafting
CT	= Conventional
IC	= Intact control
NT	= No-touch
SEM	= Scanning electron microscopy
SVG	= Saphenous vein graft
TEM	= Transmission electron microscopy

## INTRODUCTION

Although the saphenous vein graft (SVG) remains an important conduit for patients undergoing coronary artery bypass grafting (CABG), inferior patency of the SVG remains an unresolved problem. In 1996, Souza proposed the no-touch (NT) vein harvesting technique, which involves harvesting a pedicled SVG with the perivascular tissue intact without direct contact with the vein or high-pressure distension^[^1^]^ and reported improved long-term patency^[^2^-^6^]^. Concerning the improved long-term patency, previous studies suggested that the NT SVG harvesting technique preserved the morphological architecture of the luminal endothelium^[^7^-^10^]^. For example, Souza et al.^[^7^]^ reported that NT veins maintain an intact endothelium by quantifying endothelial integrity using scanning electron microscopy (SEM) and qualitatively describing the endothelial morphology using SEM and transmission electron microscopy (TEM). In that study, they suggested that mechanical distention of the saphenous vein with high-pressure saline causes endothelial cell damage. However, morphological changes in endothelial cell organelles in NT and conventional (CT) SVGs have not been fully investigated.

The CT technique certainly damages endothelial cells; however, a wide variety of endothelial damage depends on surgical technique and patient factors. Even when light microscopy showed that CT SVG endothelial cells appeared to be preserved, the observed endothelial cells could be potentially damaged during conventional preparation. Therefore, to address the potential damage at the ultrastructural level, a detailed assessment of cell organelles is essential. In this study, we assessed the morphological architecture, including cell organelles in NT and CT SVGs, compared with an intact control (IC) using TEM.

## METHODS

### Materials

This observational study was approved by the institutional review board (study approval A18-137). An SVG of the lower leg from a 71-year-old woman undergoing routine CABG was harvested with its surrounding tissue using the NT SVG harvesting technique and used for the following evaluation.

### No-Touch Saphenous Vein Graft Harvesting Technique

A NT SVG was harvested according to the technique described by Souza^[^1^]^. Briefly, preoperative ultrasonographic mapping of the saphenous vein was performed to reduce the size of dissection without unnecessary incision. Next, the SVG was dissected with its surrounding perivascular tissue intact, avoiding directly grasping the saphenous vein. High-pressure manual dilatation was avoided, and the saphenous vein was dilated gently using the patient’s arterial pressure line.

### Samples

**Fig. 1:**
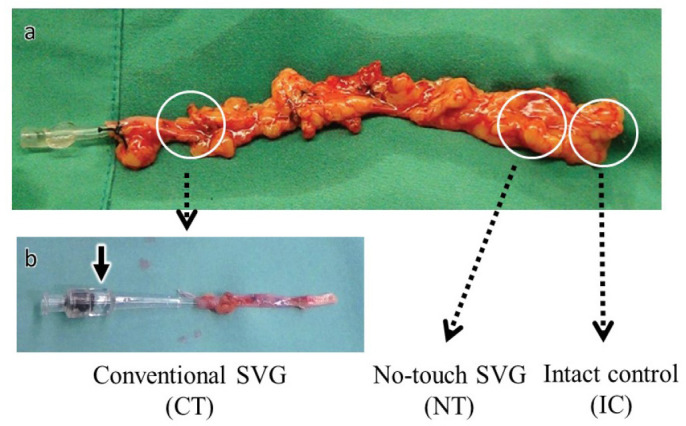
Preparing the saphenous vein graft (SVG) specimens for light and transmission electron microscopy using the two different harvesting methods (NT SVG and CT SVG) and the IC. Harvested saphenous vein was divided into three pieces and prepared using IC, NT, or CT (a). In CT (b), the surrounding tissue was removed, and the saphenous vein was distended with saline using a syringe with a cannula (arrow).

The proximal segment (1 cm) of the harvested saphenous vein with its surrounding tissue was immediately fixed before dilatation and used as the IC. The remaining saphenous vein was connected to the patient’s arterial pressure line and dilated gently using the patient’s blood pressure as described previously^[^11^]^. The proximal segment (1 cm) of the dilated saphenous vein was fixed just before CABG and used as the NT saphenous vein. The remaining part of the saphenous vein was used for grafting. The excess distal part of the saphenous vein was obtained upon completion of proximal anastomosis, stripped off its surrounding tissue, distended with saline using a syringe according to CT SVG preparation techniques, and 1 cm of this part was used as the CT following fixation (Figure 1).

Upon fixation, tissues were cut into small pieces using razor blades, and most pieces were fixed with 2.5% glutaraldehyde in a 0.1-M phosphate buffer (pH 7.4) with 4% sucrose for 2 hours at 4°C. Next, the glutaraldehyde-fixed tissues were washed using a 0.1-M phosphate buffer (pH 7.2) with 4% sucrose, fixed in 1% OsO4 in the same buffer (pH 7.4) for 90 minutes at 4°C, dehydrated in an ethanol series, and embedded in Quetol 812 epoxy resin (Nissin EM, Tokyo, Japan). Thereafter, ultrathin sections were prepared, stained using 2% uranyl acetate and Reynolds’ solution for five minutes each, and then examined using an H-7600 electron microscope (Hitachi, Tokyo, Japan). Other pieces of tissues were fixed with formalin, embedded in paraffin wax, and observed using light microscopy after sectioning and staining with conventional hematoxylin and eosin.

### Quantitative Evaluation of Organelles in Endothelial Cells

For the quantitative evaluation of organelles in endothelial cells, the size and circularity of nuclear profiles in each TEM image were measured following manual segmentation using Fiji - an open-source platform for biological-image analysis^[^12^]^. Furthermore, mitochondrial profile size in each specimen was similarly measured from the images at × 8,000~10,000 magnification.

### Statistical Analysis

Continuous variables are presented as median (first quartile; third quartile). One-way analysis of variance was used to confirm the difference between the groups and Steel-Dwass test was performed as a post-hoc analysis. All statistical analyses were performed using JMP (SAS Institute Inc., Cary, North Carolina, United States of America). *P*-values < 0.05 were considered statistically significant.

## RESULTS

### Light Microscopy Findings

**Fig. 2:**
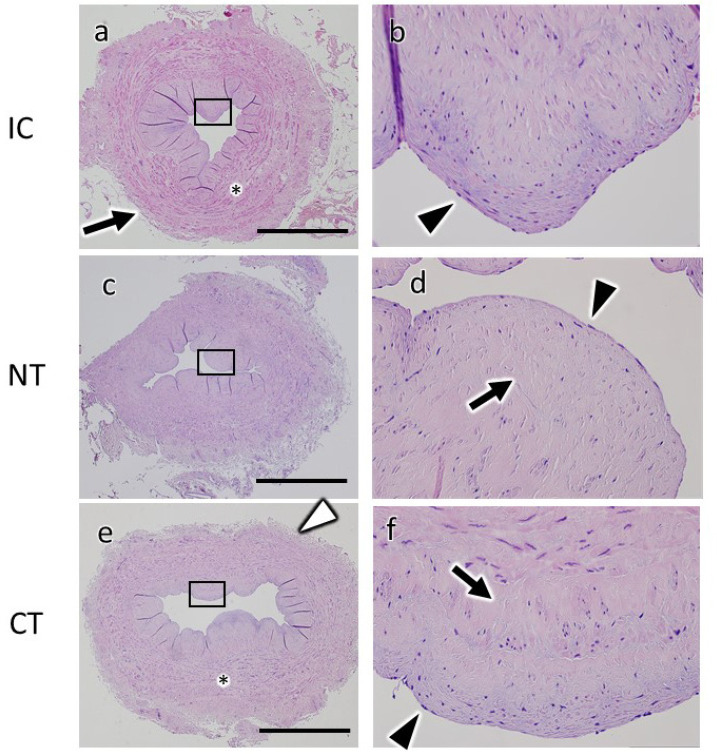
Light microscopy images. Light microscopy differences of the saphenous vein graft (SVG) between the two harvesting methods (no-touch [NT] and conventional [CT]) and intact control (IC). Light microscopy images of paraffin SVG sections prepared for the IC (a, b), NT SVG (c, d), or CT SVG (e, f) and stained with hematoxylin-eosin were acquired at low (a, c, e) and high (b, d, f) magnification. Areas marked with rectangles (a, c, e) are magnified (b, d, f). In IC (a, b), endothelial cells (b, arrowhead) and adventitia (a, arrow) were well preserved, and vascular smooth muscle (a, asterisk) was thick. In NT (c, d), most endothelial cells (d, arrowhead) were preserved, and the intima and subendothelial structures showed slight edematous changes (d, arrow). In CT (e), loss of endothelial nuclei was not obvious (f, arrowhead), and the intima and subendothelial structures showed edematous changes (f, arrow). The tunica media and smooth muscle were stretched (e, asterisk), and the adventitia was detached (e, white arrowhead). Scale bars: 1 mm. Perivascular fat tissue of NT was removed before fixation.

We first compared the gross morphological differences caused by the different harvesting techniques using light microscopy with hematoxylin and eosin staining on sections of paraffin-embedded samples. The IC showed normal saphenous vein morphology with preserved endothelial nuclei, thick vascular smooth muscle, and preserved adventitia (Figures 2A and B). A large number of the endothelial nuclei on the luminal surface of NT were preserved, and slight edematous changes were observed in the subendothelial matrix compared with IC (Figures 2C and D). In CT, the loss of endothelial nuclei was not obvious, and we observed substantial numbers of nuclei on the luminal surface of the SVG (Figures 2E and F). However, the intima and subendothelial structures showed edematous changes (Figures 2E and F). In addition, the tunica media and smooth muscle were stretched, and the adventitia was detached. These observations suggest that, while subendothelial structures were variable among different harvesting techniques, endothelial cells were comparably maintained on the luminal surface of CT as well as in NT and IC at the light microscopy level.

### Transmission Electron Microscopy Findings

Although endothelial cells appeared to be preserved on the luminal vein surface in CT, the observed endothelial cells could be potentially damaged during CT preparation. Therefore, to address the potential damage at the ultrastructural level, the following evaluation by TEM was performed.

### Intact Control (Figures 3A and B)

**Fig. 3:**
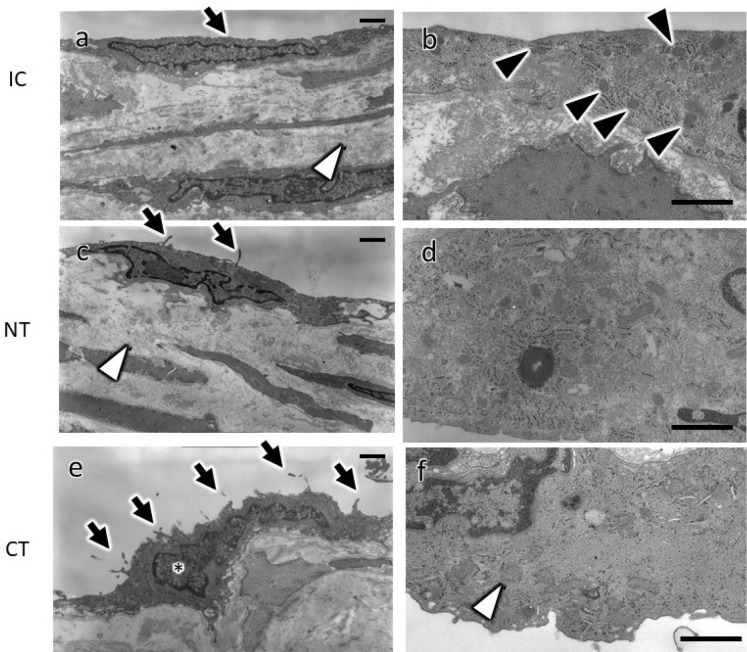
Transmission electron microscopy images of the saphenous vein graft (SVG) tissues prepared by different harvesting methods (no-touch [NT] and conventional [CT]) and the intact control (IC). The images show the IC vein (a, b), NT SVG (c, d), and CT SVG (e, f) at low (a, c, e) and high (b, d, f) magnification. In IC (a), endothelial cells had smooth and thin shapes and covered the entire luminal surface of the saphenous vein (a, arrows). Few microvilli and small vesicles were observed in the endothelial cells (a, arrows). The collagen fibers kept an orderly arrangement in the subendothelial matrix (a, white arrowhead). In the cytoplasm of endothelial cells, the mitochondria were rich, and their shapes were thin and long (b, arrowheads). In NT (c, d), the morphology of the saphenous vein was mostly preserved. The shape of many endothelial cells was similar to that in IC. However, some endothelial cells in NT showed a slight change in their surfaces, characterized by microvilli formation (c, arrows). A slight edematous change was observed in the subendothelial matrix in NT, and (the bundles of collagen fibrils were less obvious in NT) arrangement became slightly crude compared with the IC (c, white arrowhead). In CT (e, f), mitochondria in the remaining endothelial cells were enlarged and swollen (f, white arrowhead). Furthermore, their nuclei appear larger (e, asterisk). Prominent microvilli and a decreased number of vesicles were observed on the surface of endothelial cells (e, arrows). Severe edematous changes were also observed in some areas of subendothelial tissue. Scale bars: 1 µm.

In the IC, endothelial cells showed normal smooth and thin shapes and covered the whole luminal surface of the saphenous vein. The collagen fibers kept an orderly arrangement in the subendothelial matrix. Several mitochondria were observed in the endothelial cells, and their shapes were small and thin. Furthermore, a few microvilli and small vesicles were observed near the surface of the endothelial cells.

### No-Touch (Figures 3C and D)

The morphology of the endothelial cells in NT was well preserved and similar to that in IC. However, some endothelial cells showed subtle changes from those in IC, characterized by their surfaces and microvilli formation. Moreover, a slight edematous change was observed in the subendothelial matrix, and the collagen fiber arrangement was slightly irregular compared with that of IC. On the other hand, almost normal saphenous vein morphology was preserved in NT.

### Conventional (Figures 3E and F)

Although endothelial cells appeared to be maintained in substantial areas of the luminal surface in light microscopy observations, the nuclei of preserved endothelial cells were swollen, and the electron density appeared to be low. Furthermore, mitochondria in the preserved endothelial cells were enlarged and swollen. Prominent microvilli formation was observed on the surface of endothelial cells, and vesicles were not frequent in their cytoplasm. Severe edematous changes were observed in some areas of subendothelial tissue.

### Quantitative Evaluation of Organelles in Endothelial Cells by Transmission Electron Microscopy

**Fig. 4:**
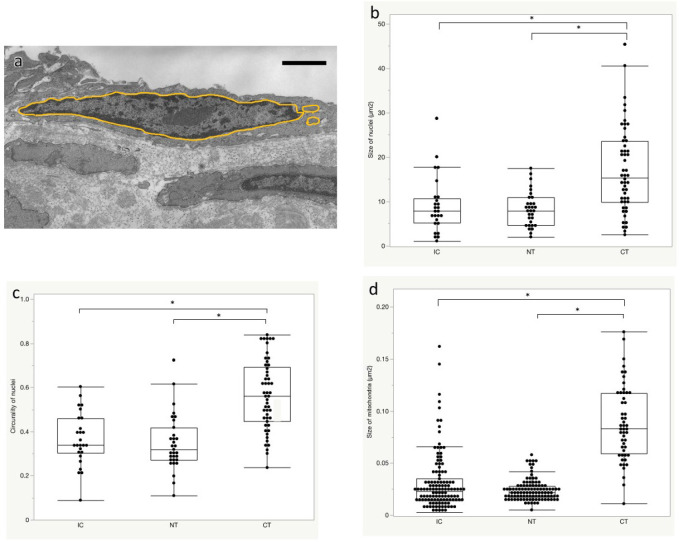
Quantitative measurements of nuclei and mitochondria in endothelial cells of the saphenous vein graft (SVG) prepared by the two different harvesting methods (no-touch [NT] and conventional [CT]) and intact control (IC). In the electron microscopy images of the IC vein, NT SVG, and CT SVG, the nuclei and mitochondria were manually segmented (a), and the size (b) and circularity (c) of the nuclei and mitochondrial sizes (d) were measured. Scale bar: 1 µm. *P < 0.05 in Steel-Dwass test. Medians with interquartile ranges and ranges of min. and max. values are shown.

To confirm the changes in nuclei and mitochondria in a larger number of cells, quantitative measurements and comparisons of the TEM images were performed by manual segmentation of the organelles (Figure 4A). The nuclear size was measured in 26 cells per 12 slides in IC, 31 cells per 11 slides in NT, and 51 cells per 20 slides in CT. The nuclear sizes were 7.21 (4.59, 10.59), 7.52 (4.88, 11.76), and 10.54 (6.68, 19.69) µm^2^ in IC, NT, and CT, respectively, and the mean nuclear size of CT was larger than those of IC and NT (CT *vs.* IC, *P*<0.0001; CT *vs.* NT, *P*=0.0002; and NT *vs.* IC, *P*=0.808) (Figure 4B). In addition, endothelial nuclei circularity was 0.34 (0.27, 0.46), 0.32 (0.25, 0.45), and 0.46 (0.31, 0.64) µm^2^ in IC, NT, and CT, respectively, and endothelial nuclei circularity in CT was higher than that in IC and NT (CT *vs.* IC, *P*<0.0001; CT *vs.* NT, *P*=0.0002; and NT *vs.* IC, *P*=0.275) (Figure 4C). Nuclei size and circularity were not significantly different between NT and IC (Figures 4B and C).

**Supplement 1:**
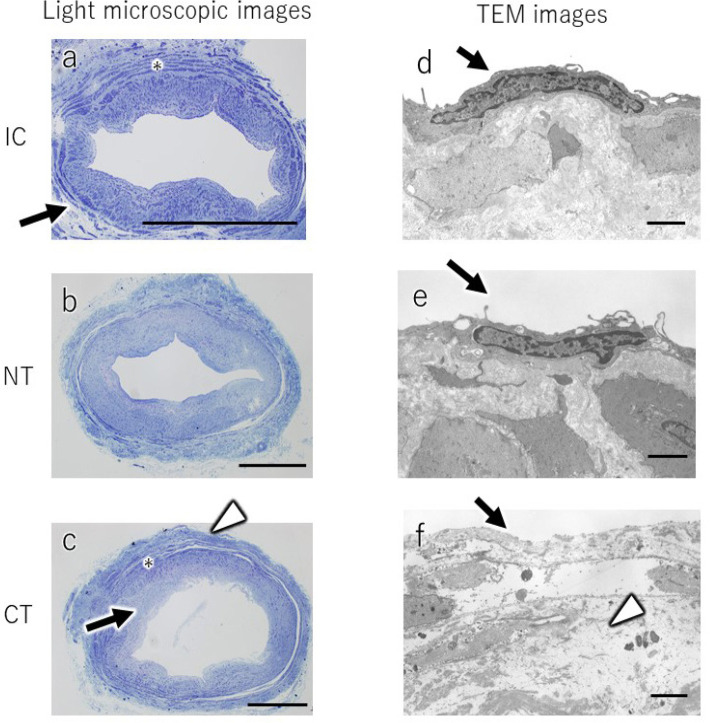
Light microscopic and transmission electron microscopy (TEM) images of resin-embedded samples prepared by the two harvesting methods (no-touch [NT] and conventional [CT]) and intact control (IC). Light microscopic images obtained from paraffin sections of the IC vein (a), NT SVG (b), and CT SVG (c) stained with toluidine-blue are shown. In IC (a), adventitia (a, arrow) is well preserved, and vascular smooth muscle (a, asterisk) is thick. In NT (b), adventitia and vascular smooth muscle show similar morphology to IC. In CT (c), the intima and subendothelial structures showed edematous changes (c, arrow). The tunica media and smooth muscle were stretched (c, asterisk), and the adventitia was detached (c, white arrowhead). Scale bars: 1 mm. TEM images of IC (d), NT (e), and CT (f). In IC (d), endothelial cells had a smooth and thin shape (d, arrow). In NT (e), the normal morphology of the saphenous vein was mostly preserved. The shape of many endothelial cells was similar to that in IC. However, some endothelial cells in NT showed a slight change in their surfaces, characterized by microvilli formation (e, arrow). In CT (f), endothelial cells were delaminated and stripped off (f, arrow). Severe edematous change was also observed in some areas of subendothelial tissue (f, white arrowhead). Scale bars: 2 µm.

Mitochondrial size assessment was also performed in 112 mitochondria per 18 slides in IC, 105 mitochondria per 17 slides in NT, and 51 mitochondria per 16 slides in CT. Mitochondrial profile sizes were 0.023 (0.014, 0.035), 0.0075 (0.0040, 0.029), and 0.083 (0.059, 0.12) µm^2^ in IC, NT, and CT, respectively, and the size in CT was larger than that in the IC and NT groups (CT *vs.* IC, *P*<0.0001; CT *vs.* NT, *P*<0.0001; and NT *vs.* IC, *P*<0.0001) (Figure 4D). These results suggest that nuclear and mitochondrial morphology in CT was disorganized even in the preserved endothelial cells, while that in NT was comparable with that in IC.

## DISCUSSION

The main findings of the present study are as follows: 1) the endothelial cells in CT SVG appeared to be preserved at the light microscopy level but are damaged at the cell organelle level, 2) the normal morphology of cell organelles was largely preserved in NT SVG.

Regarding the improved long-term patency of NT SVG, previous studies have suggested that the preserved morphological architecture of the saphenous vein contributes to long-term patency^[^7^-^10^]^. In particular, the morphological preservation of the luminal endothelium reportedly contributes to long-term patency^[^7^-^10^]^. However, there was considerable endothelial damage in the SVG harvested by the CT technique that was dependent on patient background and preparation techniques. Some CT SVGs showed severe endothelial damage (endothelial cells were almost detached; Supplement 1), while others showed preserved endothelial coverage, as shown in Figure 2.

According to the CD31 immunostaining study by Tsui et al.^[^8^]^ (2001), 30-90% of endothelial cells were preserved even if harvesting was by the CT technique. Saito et al.^[^13^]^ (2020) reported that the hyperfine structures in SEM, including microvilli and the von Willebrand factor immunostaining of the endothelial cells, were indistinguishable between CT and NT SVG. Although the endothelial cells are not often preserved in CT SVG, individual differences were observed for each graft^[^7^,^8^]^. The preservation of endothelial cells at the light microscopy level in CT SVG may be attributable to a good saphenous vein, less external damage to the saphenous vein, short pressure during dilation, etc. However, even if endothelial cell appearance in conventional TEM is comparable between different harvesting methods, organellar damage in endothelial cells is possibly triggered by conventional distension.

The current study demonstrated that nuclear size, circularity, or mitochondrial size did not significantly differ between the NT SVG and IC; however, CT SVG showed larger and more spherical nuclei and larger mitochondria than those of NT SVG and the IC. Vacuolation, such as enlarged nuclei and mitochondria, indicates cellular damage, which might lead to necrosis^[^14^-^17^]^. Therefore, the preserved endothelial cells in CT SVG may suffer substantial damage, which may compromise SVG patency. In contrast, these factors did not differ significantly between NT SVG and the IC, suggesting that endothelial cell integrity was preserved at the organelle level in NT SVG. Endothelial cell integrity is assessed by CD31 immunostaining^[^8^,^10^]^, but this technique may not represent all aspects of endothelial cell quality (*e.g.*, whether endothelial cells are alive or not, whether they maintain normal cell function, etc.). In the present study, the status of endothelial cells could be accurately evaluated by TEM at the organelle level. Nevertheless, to further assess whether cytotoxicity leads to necrosis or recovery, activation of the cascade via the tumor necrosis factor and receptor-interacting proteins 1 and 3 needs to be examined.

Several researchers have investigated gene/stem cell therapy to prevent vein graft diseases, targeting repairing/replacing damaged cells experimentally^[^10^]^. However, the NT SVG harvesting technique is more practical than gene/stem cell therapy if it preserves the normal morphological architecture of the saphenous vein without mechanical injury. Although randomized clinical trials for long-term patency are anticipated, the NT SVG harvesting technique might result in a paradigm shift in CABG strategies.

### Limitations

This study has several limitations. 1) It was based on the microscopic analysis of the IC and NT and CT SVGs from a single patient; therefore, variability attributable to individual differences should be considered. 2) The samples were obtained from different parts of the SVG (two proximal and one distal), and it might affect the histological difference between the samples. 3) The current study evaluated the ultrastructural changes in endothelial cell organelles and did not include an evaluation of other factors such as inflammatory response, random deoxyribonucleic acid degradation, and lysosomal leakage. Future studies need to evaluate these factors to clarify the relationship between morphological changes and endothelial injury culminating in necrosis or apoptosis. 4) It has been reported that the normal morphology of medial vascular smooth muscle^[^9^,^10^,^18^]^ and tunica adventitia/surrounding adipose tissue^[^8^,^10^,^13^,^19^]^ were preserved in NT SVG. Therefore, in addition to endothelial cell organelles, similar changes in medial vascular smooth muscle and adventitial and adipose tissue cells should be investigated.

## CONCLUSION

Although the endothelial cells in CT SVG appeared to be preserved under light microscopy, damage was present, and cell necrosis progressed at the cell organelle level. In contrast, NT SVG maintained healthy cell organelle morphology. These results might contribute to the improved long-term patency of NT SVG.

**Table:** 

Authors’ Roles & Responsibilities	
AS	Substantial contributions to the conception or design of the work; or the acquisition, analysis, or interpretation of data for the work; drafting the work or revising it critically for important intellectual content; agreement to be accountable for all aspects of the work in ensuring that questions related to the accuracy or integrity of any part of the work are appropriately investigated and resolved; final approval of the version to be published
NO	Substantial contributions to the conception or design of the work; or the acquisition, analysis, or interpretation of data for the work; drafting the work or revising it critically for important intellectual content; agreement to be accountable for all aspects of the work in ensuring that questions related to the accuracy or integrity of any part of the work are appropriately investigated and resolved; final approval of the version to be published
TY	Agreement to be accountable for all aspects of the work in ensuring that questions related to the accuracy or integrity of any part of the work are appropriately investigated and resolved; final approval of the version to be published
KK	Substantial contributions to the conception or design of the work; or the acquisition, analysis, or interpretation of data for the work; drafting the work or revising it critically for important intellectual content; agreement to be accountable for all aspects of the work in ensuring that questions related to the accuracy or integrity of any part of the work are appropriately investigated and resolved; final approval of the version to be published
